# How to promote adverse drug reaction reports using information systems – a systematic review and meta-analysis

**DOI:** 10.1186/s12911-016-0265-8

**Published:** 2016-03-01

**Authors:** Inês Ribeiro-Vaz, Ana-Marta Silva, Cristina Costa Santos, Ricardo Cruz-Correia

**Affiliations:** Northern Pharmacovigilance Centre, Faculty of Medicine, University of Porto, Rua Doutor Plácido da Costa, 4200-450 Porto, Portugal; Center for Health Technology and Service Research (CINTESIS), Faculty of Medicine of the University of Porto, Porto, Portugal; Health Information and Decision Sciences Department (CIDES), Faculty of Medicine of the University of Porto, Rua Doutor Plácido da Costa, 4200-450 Porto, Portugal

**Keywords:** Adverse drug reactions report, Information systems, Pharmacovigilance

## Abstract

**Background:**

Adverse drug reactions (ADRs) are a well-recognized public health problem and a major cause of death and hospitalization in developed countries. The safety of a new drug cannot be established until it has been on the market for several years. Keeping drug reactions under surveillance through pharmacovigilance systems is indispensable. However, underreporting is a major issue that undermines the effectiveness of spontaneous reports. Our work presents a systematic review on the use of information systems for the promotion of ADR reporting. The aim of this work is to describe the state of the art information systems used to promote adverse drug reaction reporting.

**Methods:**

A systematic review was performed with quantitative analysis of studies describing or evaluating the use of information systems to promote adverse drug reaction reporting. Studies with data related to the number of ADRs reported before and after each intervention and the follow-up period were included in the quantitative analysis.

**Results:**

From a total of 3865 articles, 33 articles were included in the analysis; these articles described 29 different projects. Most of the projects were on a regional scale (62 %) and were performed in a hospital context (52 %). A total of 76 % performed passive promotion of ADR reporting and used web-based software (55 %). A total of 72 % targeted healthcare professionals and 24 % were oriented to patient ADR reporting. We performed a meta-analysis of 7 of the 29 projects to calculate the aggregated measure of the ADR reporting increase, which had an overall measure of 2.1 (indicating that the interventions doubled the number of ADRs reported).

**Conclusions:**

We found that most of the projects performed passive promotion of ADR reporting (i.e., facilitating the process). They were developed in hospitals and were tailored to healthcare professionals. These interventions doubled the number of ADR reports. We believe that it would be useful to develop systems to assist healthcare professionals with completing ADR reporting within electronic health records because this approach seems to be an efficient method to increase the ADR reporting rate. When this approach is not possible, it is essential to have a tool that is easily accessible on the web to report ADRs. This tool can be promoted by sending emails or through the inclusion of direct hyperlinks on healthcare professionals’ desktops.

## Background

Adverse drug reactions (ADRs) are a well-recognized public health problem worldwide and a major cause of death and hospitalization in developed countries [1]. Rare and long-term ADRs are difficult to detect during the drug development stage. Detecting new ADRs not previously identified during clinical trials is only possible when the drug begins to be used by a large population after marketing authorization (MA). The safety of a new drug cannot be established until it has been on the market for several years [2]. As such, it is indispensable to keep drug reactions under close surveillance after commercialization through a pharmacovigilance system to continuously evaluate the drug’s safety profile. In most countries, the pharmacovigilance system is based on spontaneous ADR reports made by healthcare professionals and consumers [3]. These reports can be made using paper, telephone, e-mail or through an on-line form and consist of a description of an adverse event apparently caused by a medicine. Spontaneous ADR reporting has been described as an efficient method to detect drug safety signs [4]; however, underreporting is a major issue that undermines the effectiveness of spontaneous reports. Several studies have suggested that less than 10 % of detected ADRs are effectively reported to medicine regulatory authorities [5, 6].

Worldwide, systems using informatics to promote ADR reporting or to detect the occurrence of ADRs in healthcare institutions have been tested and used, such as computer programs that allow voluntary and automated detection of ADR [7, 8] informatics tools created to analyse clinical databases [9] or websites that actively inform healthcare professionals [10].

In addition to signal detection, information and communication technologies can also be used to encourage and facilitate reporting of suspected ADR.

In the present work, a systematic review is presented on the use of information systems in pharmacovigilance. Our main goal is to describe the state of the art information systems for the passive or active promotion of adverse drug reaction reporting.

## Methods

### Eligible studies

Studies describing or evaluating the use of information systems to promote adverse drug reaction reports were selected.

### Review team

The review team is composed of two pharmacists who are experts in pharmacovigilance (Inês Ribeiro Vaz (IV) and Ana Marta Silva (AS)) and the computer scientist Ricardo Cruz Correia (RC), who is an expert in medical informatics.

### Search methods

Studies were searched in April 2014 in the bibliographic databases. We developed a search query that included the concepts adverse drug reaction, adverse drug reaction reporting system, pharmacovigilance and information system. Only articles written in English, Portuguese or French were included. We did not establish any criteria for the publication date.

Four distinct bibliographic databases were searched: Medline (via PubMed); ISI (ISI Web of Knowledge); IEEE (IEEE Xplore) and Scopus. The query search string used in Medline® was *((ADR OR “adverse drug reaction” OR “adverse drug reactions” OR “adverse drug event” OR “adverse drug events” OR “adverse dug effect” OR “adverse drug effects”) OR “pharmacovigilance”).* A similar query was used in the other databases and was adapted to the search engine.

### Selection of studies for the review

The first selection was based on the study title and abstract (when available). Two reviewers on the review team (IV and AS) were involved in study selection and read all titles/abstracts. The study was considered eligible when at least one of the reviewers decided that the title/abstract mentioned the key concept of using information systems for ADR reporting. In cases of disagreement, a consensus meeting was held with the third reviewer (RC) to decide whether the article should be selected.

The second phase of study selection was based on the full text. The team leader (IV) reviewed each full-text article. In this stage, articles were excluded based on the following criteria: (1) the articles were only focused on medication errors; (2) the articles focused on ADR detection; (3) the articles were studies without any information system implemented; (4) the articles were studies concerning data quality; (5) the articles were studies focused on website usability; (6) the articles were only the authors’ reflections on the theme; (7) the articles were studies only related to incidents that occurred in health institutions; (8) the articles were studies concerning signal detection and (9) the articles were studies concerning electronic transmission between the authority and other institutions (pharmaceutical companies or regional pharmacovigilance centres).

The articles remaining after this review were included in the final statistical analysis.

**Fig. 1:**
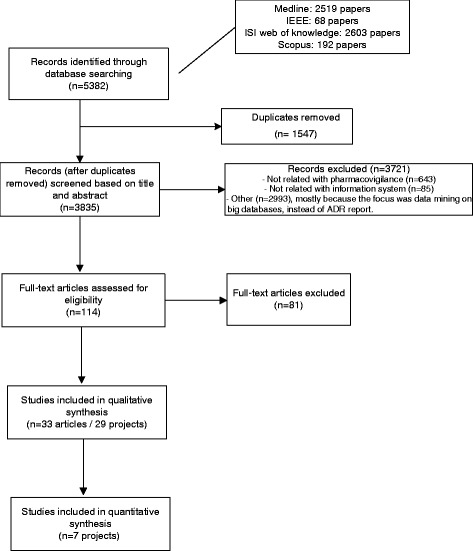
Flowchart of the study selection

These articles were grouped into research projects to avoid the distortion created by multiple papers describing the same project (Fig. 1). All statistical analyses were based on the projects and not on the articles.

### Definition of variables

The variables examined in these reviews were related to the projects, papers and information systems described in each project.

We used the following data for project identification: (1) project number; (2) Information system name (if any); (3) country; (4) publication date; (5) type of study and (6) reference(s).

According to the description of the projects, the following variables were analysed:

1. Area covered by the project (i.e., region, country, or hospital)
2. Type of action promoted by the project (passive promotion of ADR reporting or active promotion of ADR reporting)
3. Type of software (i.e., web-based or mobile)
4. Type of institution (i.e., regulatory authority or universities)
5. Target (healthcare professionals or patients)
6. Type of medicine (all, vaccines, chemotherapy, or others)
7. Type of ADR (all/serious ADRs based on the World Health Organization seriousness criteria [11])

### Statistical analysis

The inclusion criteria for the quantitative analysis were the availability of data related to the number of ADRs reported before and after each intervention and a follow-up period.

Studies that only disclosed the increased ADR rate and studies that reported zero ADRs before the project implementation were excluded because it was not possible to perform the analysis in these cases.

For each study with available data, the rate of ADRs reporting increase (quotient between ADR reports after and ADR reports before) and the respective 95 % confidence intervals were calculated. A rate of ADR reporting increase equal to 2 indicated that the ADR reports doubled after the intervention. Conversely, a rate of ADR reporting increase equal to 1 indicated that the number of ADR reports after the intervention was equal to the number of ADR reports before the intervention. The aggregated rate of the ADR reporting increase was calculated with the inverse variance method using a random effects model and a forest plot was presented. The confidence intervals, aggregated rate of ADRs and forest plot were performed using a Microsoft Excel spreadsheet. The description of the Microsoft Excel spreadsheet and the respective statistical methods used were described by Neyeloff [12].

## Results

Our search method found 2519 articles in PubMed, 68 in IEEE, 2603 in ISI and 192 in Scopus. After eliminating duplicate articles, 3835 articles were selected.

Two reviewers (IV and AS) read all 3835 titles/abstracts. In cases of disagreement, which occurred with 151 articles, a consensus meeting was held with the third reviewer (RC) to decide whether the article should be selected.

A total of 643 studies were excluded because they were not related to pharmacovigilance, 85 were excluded because they were not related to information systems and 2993 were excluded for other reasons (mostly because their focus was on data mining in large databases instead of ADR reporting).

A total of 114 of the 3835 articles were selected in this first selection based on the title and abstract.

The team leader (IV) reviewed each of the 114 full-text articles. After this review, 33 articles remained for the final statistical analysis. At this stage, most of the articles were excluded because: (1) they were only related to medication errors; (2) they were focused on ADR detection; (3) they were studies without any information system implemented; (4) they were studies concerning data quality; (5) they were studies focused on website usability; (6) they were only authors’ reflections on the theme; (7) they were studies only related to incidents that occurred at health institutions; (8) they were studies concerning signal detection or (9) they were studies concerning electronic transmission between the authority and other institutions (pharmaceutical companies or regional pharmacovigilance centres).

These 33 articles were grouped into 29 distinct research projects to avoid the distortion created by multiple papers describing the same project (Fig. 1.). All statistical analyses was based on projects and not on articles.

Table 1 lists all 29 projects, their country, the number of publications, the publication year and the journal. The country with the most published projects was the USA (11), followed by the United Kingdom (3).

**Table 1:** Project identification

Project number	System name (if any)	Country	Number of publications	Publication date(s)	References	Journals
4		USA	1	1992	[32]	Hospital pharmacy
28		France	1	2001	[26]	Fundamental & Clinical Pharmacology
11		Japan	1	2002	[33]	Yakugaku Zasshi-Journal of the Pharmaceutical Society of Japan
17		USA	1	2004	[34]	American Journal of Health-System Pharmacy
2		USA	3	2005, 2007	[25, 35, 36]	Journal of Clinical Oncology, Journal of American Medical Information Association
22		USA	2	2005, 2006	[28, 37]	Biosecurity and Bioterrorism-Biodefense Strategy Practice and Science, Health Expectations
10		USA	1	2007	[29]	Journal of the American Medical Informatics Association
9	MEADERS	USA	2	2007, 2010	[23, 38]	Annals of Family Medicine, AMIA Annual Symposium proceedings
21		Spain	1	2008	[39]	Annals of Pharmacotherapy
7		Sweden	1	2009	[15]	European Journal of Clinical Pharmacology
5		Canada	1	2010	[40]	International Journal of Medical Informatics
13		Canada	1	2010	[27]	Vaccine
18		USA	1	2010	[41]	Pharmacoepidemiology and Drug Safety
19		United Kingdom	1	2010	[42]	Archives of Disease in Childhood
23	ALIAS	USA	1	2010	[43]	Contemporary Clinical Trials
27		Taiwan	1	2010	[14]	Value in Health
8		United Kingdom	1	2011	[44]	Journal of Psychiatric and Mental Health Nursing
12		Serbia	1	2011	[20]	Drug Safety
14		France	1	2011	[13]	Therapie
15		USA	1	2011	[21]	Paediatrics
6		United Kingdom	1	2012	[45]	Drug Safety
16		Korea	1	2012	[46]	Yonsei Medical Journal.
20		Portugal	1	2012	[16]	Drug Safety
25		USA	1	2012	[47]	2012 Ninth International Conference on Information Technology: New Generations
1		Cambodge	1	2013	[22]	Journal of Medical Internet Research
3		Netherlands	1	2013	[18]	Studies in health technology and informatics
24	SALUS	France	1	2013	[48]	Studies in health technology and informatics
26		Spain	1	2013	[49]	International Journal of Clinical Pharmacy
29		Denmark	1	2013	[17]	European Journal of Hospital Pharmacy-Science and Practice

### Trends

**Fig. 2:**
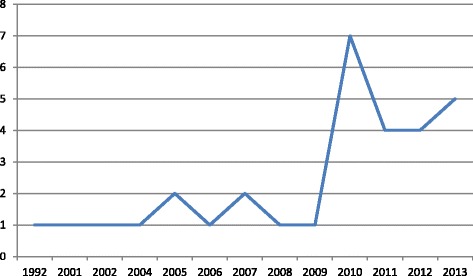
Number of publications by year

There was an increasing trend in publication, especially after 2009 (Fig. 2).

#### Qualitative analysis

The qualitative variables analysed in each project are listed in Table 2 and described below. Globally, we found that there was an increase in the publication of projects over the study period, with 4 projects published before 2001, 4 projects between 2005 and 2007, 8 projects between 2008 and 2010 and 13 projects between 2011 and 2013.

**Table 2:** Qualitative analysis of the projects

Variable	Time period				Total (%)	Project numbers
	<2004 (4 projects)	2005–2007 (4 projects)	2008–2010 (8 projects)	2011–2013 (13 projects)		
Geographic area covered by the project						
Regional	4	3	5	6	18 (62)	2, 4, 5, 7, 8, 10, 11, 13, 14, 16, 17, 20, 21, 22, 26, 27, 28, 29
National	0	1	3	6	10 (34)	1, 3, 6, 9, 15, 18, 19, 23, 24, 25
International	0	0	0	1	1 (3)	12
Area covered by the project						
Hospital	4	2	5	4	15 (52)	2, 4, 5, 8, 11, 13, 17, 18, 20, 21, 22, 26, 27, 28, 29
Community	0	0	0	6	6 (21)	1, 3, 12, 14, 15, 16
Primary care	0	2	1	1	4 (14)	6, 7, 9, 10
Other healthcare institutions *(different from hospitals or primary care)*	0	0	1	2	3 (10)	19, 24, 25
Clinical trials	0	0	1	0	1 (3)	23
Type of action promoted by the project						
Passive promotion of ADR reporting	3	3	6	10	22 (76)	4, 5, 6, 8, 9, 10, 11, 14, 15, 16, 17, 18, 19, 20, 21, 22, 23, 24, 25, 26, 27, 29
Active promotion of ADR reporting	1	1	2	3	7 (24)	1, 2, 3, 7, 12, 13, 28
Type of software						
Web-based	1	3	6	6	16 (55)	2, 3, 5, 7, 9, 12, 13, 14, 15, 16, 19, 20, 22, 23, 27, 28
System inside the Electronic Health Record	3	1	2	6	12 (41)	4, 6, 8, 10, 11, 17, 21, 24, 25, 26, 18, 29
Mobile	0	0	0	1	1 (3)	1
Type of institution promoting the study						
Hospital	2	1	3	3	9 (31)	2, 4, 8, 17, 18, 21, 26, 27, 29
University	1	1	3	4	9 (31)	5, 10, 11, 12, 13, 19, 20, 24, 25
National institution	0	2	0	2	4 (14)	1, 9, 15, 22
Regulatory authority	1	0	1	3	5 (17)	6, 7, 14, 16, 28
Other^a^	0	0	1	1	2 (7)	3, 23
Target						
Healthcare professionals	4	2	7	8	21 (72)	4, 6, 7, 8, 9, 10, 11, 13, 14, 16, 17, 18, 19, 21, 23, 24, 25, 26, 27, 28, 29.
Patients	0	2	1	4	7 (24)	1, 2, 3, 5, 12, 20, 22
Healthcare professionals and patients	0	0	0	1	1 (3)	15
Type of medicine						
All	4	1	5	11	21 (72)	3, 4, 5, 6, 7, 8, 9, 11, 12, 14, 16, 17, 18, 19, 20, 21, 24, 25, 26, 28, 29
Vaccines	0	2	1	2	5 (17)	1, 10, 13, 15, 22
Chemotherapy	0	1	0	0	1 (3)	2
Human albumin	0	0	1	0	1 (3)	23
Radiopaque agents	0	0	1	0	1 (3)	27
Type of ADR						
All	4	4	8	11	27 (93)	2, 4, 5, 6, 7, 8, 9, 10, 11, 12, 13, 14, 15, 16, 17, 18, 19, 20, 21, 22, 23, 24, 25, 26, 27, 28, 29
Serious	0	0	0	2	2 (7)	1, 3

^a^ Other institutions are: Clinical trial team (project 23) and website producer (project 3)

### Geographic area covered by the projects

Most of the projects were regional (62 %), followed by national projects (34 %). We found only 1 international project based on Facebook®. This international project was developed in the last time period (2011–2013).

### Areas covered by the projects

Most of the projects (52 %) were developed in hospitals, followed by community projects (21 %). A total of 14 % covered primary care institutions and 10 % (3 projects) were developed for use in any type of healthcare institution. One project was dedicated to a multicentre clinical trial. We also found that all of the projects oriented to the community were developed in the last 3 years (2011–2013).

### Types of actions promoted by the projects

The majority of the projects passively promoted ADR reporting (76 %); the remainder actively promoted reporting (24 %).

### Types of software

More than half of the projects (55 %) used web-based technology and 41 % used electronic health records. Only one project used mobile phone technology. There was an increasing trend in software using web-based technology over all of the time intervals considered. The mobile technology appeared during the last time period.

### Types of institutions promoting the studies

Most of the projects were promoted by hospitals and universities (31 % e*x aequo*). There were 4 projects developed by national institutions (not regulatory) and 5 projects implemented by regulatory authorities.

### Targets

A total of 72 % of the projects were geared to healthcare professionals, 24 % to patients and one project was geared to both targets. Most of the projects targeting patient ADR reporting were developed in the last years considered (2011–2013).

### Types of medicine

Most of the projects (72 %) covered all medicines, but 17 % were specific to vaccines. There were also projects specific to reporting ADRs due to chemotherapy, human albumin and radiopaque agents (1 project for each of these medicines).

### Types of ADR

Only a small percentage of the projects were specific for serious adverse drug reactions. The majority (93 %) covered all ADR.

#### Quantitative analysis

From the 29 projects analysed, seven projects met the criteria for inclusion in the quantitative analysis (meta-analysis). The criteria used were the availability of data related to the number of ADR reported before and after each intervention and a follow-up period.

From the seven projects included in the quantitative analysis, six had the same follow-up period (12 months) and only one (project 14) differed on this item (18 months of follow-up). The results are described in Table 3.

**Table 3:** Intervention effect on ADR reporting increase

Study	ADR reports before	ADR reports after	Rate	CI lower	CI upper
Project 14	287	415	1,44	−0,18	3,07
Project 7	89	111	1,25	−0,51	3,00
Project 29	30	162	5,4	4,56	6,24
Project 6	3279	4716	1,44	−0,20	3,072
Project 17	118	294	2,49	1,25	3,73
Project 27	20	62	3,1	1,99	4,21
Project 20	82	212	2,58	1,37	3,80

**Fig. 3:**
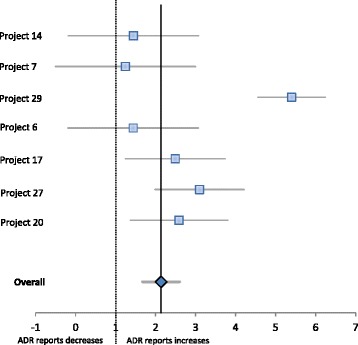
Rate (ADR reports after/ADR reports before) of ADR report increase (95 % IC)

We performed a meta-analysis with these seven projects to calculate the aggregated measure of the ADR reporting increase. The overall measure was 2.1, which indicated that the interventions performed in the analysed projects doubled the number of ADR reports (Fig. 3).

Projects 14, 7 and 6, which had similar ADR reporting increases, used different approaches. The authors of project 14 assessed an online ADR reporting form, in project 7 the authors send repeated e-mails with ADR information to healthcare professionals and project 6 evaluated the inclusion of a reporting system inside the clinical information system.

Four of the information systems that contributed to the improvement of ADR reporting used web-based technology. Two used an online reporting form (Project 14 [13] and Project 27 [14]) to facilitate ADR reporting. A Swedish group opted to evaluate the effect of repeated emails to health care professionals that contained attached ADR information (Project 7 [15]). A Portuguese study tested the inclusion of hyperlinks to the online ADR reporting form on hospitals’ electronic patient records (Project 20 [16]) to facilitate access to the ADR form.

Three projects explored the use of electronic health records to directly report the ADRs (projects 29, 6 and 17). Among these, project 29 [17], which had the best result in terms of the ADR reporting increase, was a system that completed the ADR report whenever a physician required assistance.

## Discussion

Although a limited number of projects was included in our work (n = 29), our data suggest that the number of projects that aimed to promote ADR reporting using information technologies increased over time.

Study selection was performed as a manual review; this approach caused a huge workload because we obtained more than 3000 articles. An optimized query would reduce the workload but lose sensitivity.

As expected, most of the projects that aimed to promote adverse drug reactions reporting were developed in hospitals and tailored to healthcare professionals. In fact, most of the serious ADRs were detected in hospitals and reported by healthcare professionals [18]. For example, in Europe direct reporting (ADRs reported by patients) has only been allowed for every country since 2012 [19]. This finding may also explain why most of the projects that targeted direct ADR reporting were developed in the last 3 years of the study period (2011–2013) [18, 20–22].

Most of the authors chose to develop systems for the passive promotion of ADR reporting because busy healthcare professionals only submit their suspected ADR if it does not increase their workload [23, 24]. Active promotion of ADR reporting is difficult and not always ethically acceptable because no material reward can be given to the reporters. Thus, projects that aimed to actively promote ADR reporting involved teaching sessions [25] or e-mails containing ADR information [15, 18, 26, 27].

Our results suggested that there was an increasing trend in the use of web-based software to promote ADR reporting, which could be explained by the dissemination of internet use. Nevertheless, mobile technology was also appearing.

Most of the retrieved projects covered all medicines and ADR, whereas only a few were specific. However, we found 5 projects dedicated to vaccine adverse reactions [21, 22, 27–29] and in the last 3 years two projects were developed to specifically report serious ADR [18, 22].

The institutions that primarily promoted this work were universities and hospitals because universities have the know-how to perform these actions and hospitals have specific needs to be solved. However, regulatory authorities have been increasing their involvement in the development of this type of project.

A limitation of this study is that a grey literature search was not performed. However, we think that this lack does not cause a large bias because regulatory authorities are less likely to produce this type of project. When regulatory authorities are involved in projects of this scope, they usually associate with universities and hospitals that have a greater incentive to publish.

Based on our quantitative analysis, we can conclude that all of the projects analysed increased the ADR reporting numbers (most by approximately two-fold). We found two projects that increased ADR reporting by more than two-fold [14, 17], perhaps because their basal values were much lower compared with the other five projects. A similar effect was noted previously in two other studies when the same population of health care professionals was exposed to the same educational interventions two different times [30, 31]. After the first intervention, the authors achieved a much higher effect and ADR reporting increased compared to the second intervention due to the differences in the initial values.

In our quantitative analysis, we found a limitation concerning the aggregation of the information because we found only 7 studies that provided data concerning its impact on the increase in ADR reporting. These data were not available for the other studies even after we contacted the authors. However, we did not identify any variable that could distinguish these 7 projects from the other 22 projects. We must reiterate the importance of providing quantitative data when publishing studies focused on interventions that aim to promote ADR reporting.

Worldwide underreporting of ADR is a major concern, and many institutions are aware that it is feasible to use information systems to improve ADR reporting. The most commonly used platform is web-based and exhibits an increasing trend, but interventions inside electronic health records also have the potential to improve pharmacovigilance activities and particularly ADR reporting. Direct ADR reporting is being increasingly taken into account when the aim is to improve information on drug safety.

Based on our results, we believe that it would be useful to adopt a system to assist healthcare professionals with completing ADR reporting within electronic health records because this approach seems to be an efficient method to increase the ADR reporting rate. When this approach is not possible, it is essential to have a tool that is easily accessible on the web to report ADR. This tool can be promoted by sending emails or through the inclusion of direct hyperlinks on healthcare professionals’ desktops.

## Conclusions

Our systematic review allowed us to note some facts about interventions that aim to improve ADR reporting using information systems. According to our aggregation analysis, these interventions doubled the number of ADR reports. We also found that most projects passively promoted ADR reporting (facilitating the reporting process) and the countries involved in this type of project were Northern America countries (USA and Canada), European countries and in a smaller number Far East countries.
